# No effect of targeted memory reactivation during sleep on retention of vocabulary in adolescents

**DOI:** 10.1038/s41598-020-61183-z

**Published:** 2020-03-06

**Authors:** Ines Wilhelm, Thomas Schreiner, Jonas Beck, Björn Rasch

**Affiliations:** 1 Department of Psychiatry and Psychotherapy, Translational Psychiatry Unit (TPU), University of Lübeck, Lübeck, Germany; 2 Ludwig Maximilians University, Department of Psychology, Cognitive Neuropsychology, Munich, Germany; 3 Department of Psychology, University of Fribourg, Fribourg, Switzerland

**Keywords:** Language, Consolidation

## Abstract

Re-exposure of newly acquired vocabulary during sleep improves later memory recall in healthy adults. The success of targeted memory reactivation (TMR) during sleep presumably depends on the presence of slow oscillations (i.e., EEG activity at a frequency of about 0.75 Hz). As slow oscillating activity is at its maximum during adolescence, we hypothesized that TMR is even more beneficial at this developmental stage. In the present study, adolescents aged 11 to 13 learnt Dutch vocabulary in the evening and were tested on recall performance the next morning. Half of the words were presented via loudspeakers during post-learning periods of NREM (Non Rapid Eye Movement) sleep in order to stimulate memory reactivation. Unexpectedly, TMR during sleep did not improve memory on the behavioral level in adolescents. On the oscillatory level, successful reactivation during sleep resulted in the characteristic increase in theta power over frontal brain regions, as reported in adults. However, we observed no increase in spindle power during successful reactivation. Possible factors that may explain the lacking effect of TMR in adolescents in this study such as differences in learning abilities and pre-sleep performance levels are discussed.

## Introduction

Second language learning essentially depends on the acquisition of novel word meanings and word forms. This newly acquired vocabulary needs to be stored for a longer period of time in order to be accessible later on e.g. when engaging in conversations, during reading or in other contexts. More specifically, the acquisition of new vocabulary initially results in the formation of a highly labile memory trace that needs to be transformed into a stable long-lasting form^1^. This process, which is also referred to as memory consolidation, has been found to benefit from periods of sleep after learning^2,3^. Sleep-dependent consolidation of memories critically relies on the repeated reactivation of newly acquired memories during sleep^2^. Such processes of memory reactivation can be boosted by presenting external memory cues of the learnt information during post-learning periods of Non-rapid-eye-movement (NREM) sleep^4–6^. Three recent studies from our group indicated that this method, also known as « targeted memory reactivation » (TMR), can affect vocabulary learning in adults^7–9^. In these studies, participants learnt a list of Dutch vocabulary and their German translation. Half of the Dutch words were presented again via loudspeaker during NREM sleep in the night after learning. In a later recall test, vocabulary that had been presented during sleep as compared to vocabulary that had not been presented was better remembered. The successful reactivation of vocabulary during sleep was found to coincide with an increase in spindle activity (i.e., EEG activity at a frequency of 10–15 Hz) and theta activity (i.e. 5–8 Hz)^7^. Previous studies had further pointed towards a crucial role of slow oscillatory activity (i.e., around 0.75 Hz) in the process of memory consolidation^10–12^. Based on the background of these findings it was recently hypothesized that slow oscillations represent an essential prerequisite for the success of targeted memory reactivation while theta activity reflects the reinstatement of the original memory trace. Furthermore, spindle activity has been related to the integration of the new memory trace into the neocortical long-term network of preexisting information and its stabilization^13^.

Slow oscillating EEG activity during sleep is at its maximum during late childhood/early adolescence (i.e., around 8–12 yrs)^14–16^ which raises the possibility that sleep is even more beneficial to the processes of memory consolidation during this developmental period^17,18^. However, empirical evidence especially in the context of language learning is scarce. Initially, studies exposed children and adults to novel word forms or novel word-object pairings and tested memory performance on these stimuli after retention intervals of 24 h and/or 1 week later. In these studies, children showed a greater improvement over time in a recognition memory test^19–21^ as well as in a cued recall test^20,22^ (see^21^ for greater improvements in cued recall performance in adults) compared to adults. Overnight improvement in recall performance was positively correlated with fast spindle density (i.e., 13.5–15 Hz) in children^22^. However, none of these studies comprised a wake control group, i.e., a group of subjects who do not sleep during the retention interval. The question, namely whether the reported differences critically rely on processes of memory consolidation during sleep or are a consequence of an increased plasticity of the developing brain independent of sleep, therefore remains open. A wake control group requires subjects to refrain from sleeping for the period of the retention interval, which raises ethical concerns especially in a children or adolescent sample. Another elegant way to learn more about sleep-dependent processes of language learning during early periods of development is to manipulate the processes that underlie the consolidation of memories during sleep, i.e., the reactivation of the memories themselves. Thus, in the present study, we used the above-mentioned method of targeted memory reactivation to study (1) the role of memory reactivation during nocturnal sleep on the consolidation of newly acquired vocabulary as indicated by retention performance the next morning and one week later and (2) the neuronal correlates of reactivating newly acquired vocabulary during nocturnal sleep, in 11–13 year-old participants.

## Methods

### Subjects

Nineteen healthy adolescents aged between 11 and 13 years were recruited via advertisements placed at the children’s hospital. Subjects were monolingual or bilingual. Note that the sample size was determined as a consequence of a power analysis using GPower^23^. The estimation of effect size was based on our previous studies on vocabulary learning in adults, which used a comparable within-subjects design reporting large effect sizes of d = 0.8^8,24^. According to this power analysis a sample size of n = 15 would be required to find similar large effects with a power of 0.8. Interviews as well as standardized questionnaires ensured that the participants had no behavioral problems, cognitive impairments or sleep disorders. In order to exclude any psychiatric diagnosis, a clinical interview was performed using the Mini-International Neuropsychiatric Interview (M.I.N.I.-KID)^25^. Only participants with an IQ between 85 and 135 as indicated by the Wechsler-Intelligence Scale for Children (WISC-IV)^26^ were included in this experiment. Participants did not take any medication at the time of the experiment, and the ingestion of caffeine or alcohol was not allowed on experimental days. Only participants who spoke German as a first language without any prior Dutch language skills were included. All participants were asked for individual sleep habits, i.e., usual time to go to bed, time getting up, etc. This information was used to schedule both nights in the sleep laboratory in accordance with their usual sleep habits. Also, participants noted their daily activities regarding sleep and waking times in a sleep diary for the seven days prior to the experiment. This data was additionally compared to, and supplemented with, actigraphical monitoring. The study was approved by the local ethics committee (Ethics Committee of the Philosophical Faculty of the University of Zurich) and procedures were carried out in accordance with the approved guidelines. Participants as well as their parents gave written informed consent before participating. One participant had to be excluded due to poor sleep quality in the experimental night (>15% time awake). The remaining 18 participants (nine males, nine females; *M* = 12.66, *SD* = 1.12) were included in the analysis of the behavioral data. For the EEG analyses (final n = 14), four additional participants had to be excluded: two due to technical problems with the EEG recordings and another two due to an insufficient amount (<7) of gains (i.e., Dutch words not remembered before sleep but correctly recalled after sleep) or losses (i.e., words correctly retrieved before sleep but not remembered after sleep).

### Design and procedure

**Figure 1:**
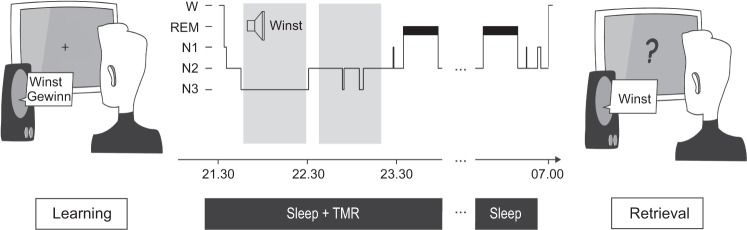
Experimental procedure. After studying 92 Dutch-German word pairs in the evening, participants slept a whole night. During subsequent NREM sleep, half of the learnt Dutch words were presented again. Half of the cued words consisted of previously correct remembered words (i.e., hits) whereas the other half was taken from unknown words (i.e., misses). Words were presented every 2.8–3.2 s for at least 45 minutes. A cued recall procedure was conducted after sleep to test the participant’s memory for the German translations.

The general procedure was comparable to our previous study in young adults^8^ (Please see Fig. 1 for an illustration of the design and procedure). Participants were familiarized with the procedure during an adaptation night preceding the experimental night, which allowed them to get used to polysomnographic recordings. Experimental and adaptation nights were separated by at least one night at home to exclude possible effects of reduced sleep quality during the adaptation night on sleep in the experimental night. During the experimental night, participants arrived at the sleep lab around 2 h before their habitual bedtime (around 7 PM). First, an electrode net was fitted over the participants’ head in order to record nocturnal sleep during the subsequent night. Once the net was in place, the participants performed the vocabulary learning task (Learning Session) and went to bed afterwards. During NREM sleep, half of the learnt words were presented via a loudspeaker that were placed behind the participants’ head. This procedure is assumed to stimulate the reactivation of the German translation of the respective the Dutch words they had learned during the learning session. Testing started ~45 minutes after waking up in the morning to avoid any impact of sleep inertia on recall performance. An additional test (Test Session 2) was performed one week later in order to test possible long-term effect of memory cueing.

### Sleep EEG

In both the experimental and the adaptation nights, sleep was recorded using a high-density sleep EEG (Electrical Geodesics Sensor Net for long-term monitoring, 128 channels, referenced to a vertex electrode) which also included electromyographic and electrooculographic recordings. Data were sampled at 500 Hz. Furthermore, based on the standard criteria provided by the American Academy of Sleep Medicine^27^, EEG data was manually scored for sleep stages N1, N2, N3 as well as Wake and REM sleep at frontal, central and occipital electrodes over 30 s epochs. See Table 1 for descriptive statistics of the sleep parameters.

**Table 1:** Sleep Parameters.

Sleep parameter	Mean ± SEM
WASO (min)	26.31 ± 5.58
N1 (min)	24.69 ± 2.66
N2 (min)	253.25 ± 15.28
N3 (min)	138.83 ± 10.78
REM (min)	96.25 ± 8.70
SPT (min)	539.78 ± 16.42
% WASO	4.83 ± 0.99
% N1	4.56 ± 0.44
% N2	46.68 ± 2.07
% N3	26.18 ± 2.13
% REM	17.68 ± 1.45

*Note*. Means in % ± SEM relative sleep period time and min ± SEM. Given is the time spent awake after sleep onset (WASO), in sleep stage 1 (N1), sleep stage 2 (N2), sleep stage 3 (N3), rapid eye movement (REM) sleep and Sleep Period Time (SPT) including WASO.

### Vocabulary learning task

Based on the results of a pilot study conducted on four young participants, including children and adolescents (age: *M* = 12.69, *SD* = 3.34), we decided to reduce the number of Dutch-German word pairs from 120 to 92 for this age group in order to reach pre-sleep levels of performance that were comparable to those found in our previous studies in adults, i.e., 50–60%^8^. The words were presented in a random order in three rounds. Dutch words were presented acoustically (duration range: 400–650 ms) via loudspeaker (about 70 dB sound pressure level). During the first learning block each Dutch word was succeeded by a visual presentation of its German translation (2000 ms). The inter-trial interval between consecutive word pairs was 2000–2200 ms. The participants were instructed to memorize as many translations as possible and were informed that they would be tested at a later time. In the second round the Dutch words were presented again followed by a question mark indicating that the participants task was to vocalize the corresponding German word. Independent from the correctness of the participants´ response, the correct German word was displayed again for 2000 ms. In the third round, as well as in the recall session the next morning, the cued recall procedure was repeated without any feedback of the correct German translation. The answers given in the third learning round served as the pre-sleep baseline values. In five participants, one additional round with feedback was introduced before the final round without feedback in order to increase the learning level.

### Targeted memory reactivation during sleep

During post-learning periods of NREM sleep, Dutch words were presented auditorily via loudspeaker (with a 55 dB sound pressure level). Half of the learnt words (i.e., 46 words) were cued, whereas the other half of the learnt words were not presented during sleep. Half of the cued words were randomly and individually chosen from the group of words that had been correctly remembered by an individual during the learning phase (i.e., hits), whereas the other half was taken from incorrectly remembered words (i.e., misses). Words were presented every 2.800–3.200 s in a randomized order for a total of at least 45 minutes (see Table 2 for details about word presentation). An experienced experimenter inspected the EEG in real-time to determine sleep stages and to detect any indication of arousal. Word cueing was started when a participant spent 10 minutes in stage N3 and proceeded as long as the participant remained in N2 or N3 sleep. Cue presentation was immediately stopped whenever any sign of an arousal, movement, wakefulness or change to stage N1 or REM sleep was observed by the experimenter. After reoccurring N3 sleep, cue presentation was continued.

**Table 2:** Presentation of words during the night.

Parameter	Range	Mean ± SEM
Duration of cueing (min)	49–84	65.22 ± 2.07
Stops during reactivation	2–15	6.44 ± 0.84

*Note*. Range (min – max) and means ± SEM of all participants.

### Analyses of power changes in response to cues

EEG data analysis included four participants with one additional learning round with feedback in the vocabulary learning task before sleep. Please note, that EEG data analysis in this study is similar to data analysis performed in our previous experiment in adults (see^8^ for further information on EEG analysis). EEG signals were preprocessed using Brain Vision Analyzer 2.0 (Brain Products, Gilching, Germany). Preprocessing included re-referencing the raw EEG data to the average of the two mastoids as well as low-pass (30 Hz, roll-off 24 dB per octave) and high-pass filtering (0.1 Hz, roll-off 12 dB per octave). EEG data were epoched into segments which included 2 s before and 3 s after word onset. Similar to previous studies, segments were categorized in “Gain” words (i.e., Dutch words not remembered before sleep but correctly recalled after sleep), “Loss” words (i.e., words correctly retrieved before sleep but not remembered after sleep), “HitHit” words (i.e., words correctly retrieved before and after sleep) and “MissMiss” words (i.e., words not remembered before and after sleep)^7^. Additional EEG oscillatory analyses were performed using the Fieldtrip toolbox^28^ running on Matlab R2014a (MathWorks, Natick, MA). Time-frequency analysis was computed trial by trial using a 7-cycle Morlet wavelet decomposition (2 to 25 Hz, step size of 0.5 Hz). A sliding window (step size of 10 ms) was applied across the entire length of the epochs. Single trials were normalized with respect to a pre-stimulus time window ranging from −1000 ms to −100 ms.

### Statistical analyses

Statistical analyses were based on student’s t-tests. The level of significance was set to *p* ≤ 0.05. For correlational analyses, Kendall’s Tau was used due to our small sample size. Statistical quantification of the EEG data was accomplished using a cluster-based nonparametric permutation approach in FieldTrip^29^. Initially, oscillatory power in the theta (6–8 Hz) and sleep spindle (12–16 Hz) range were averaged over frequency and differences between gain and loss trials were tested using paired sample t-tests (*p* < 0.05; two-tailed). To correct for multiple comparisons, 1000 permutations were drawn and the cluster with the largest summed t-value was tested against the permutation distribution.

## Results

### Learning and retention performance

**Figure 2:**
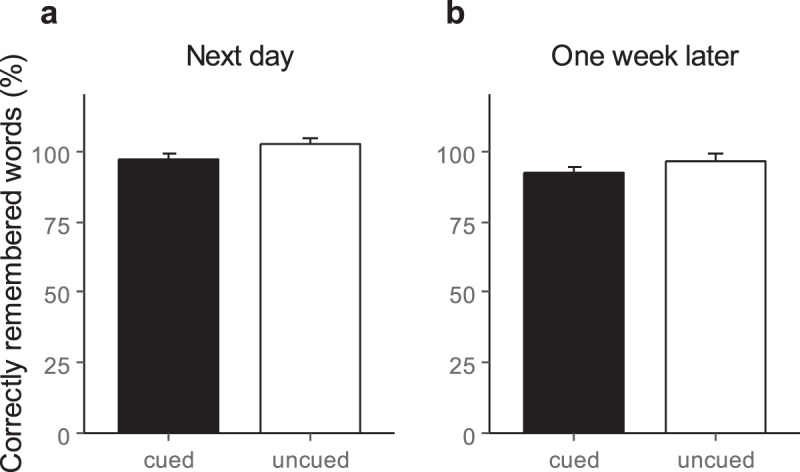
Memory performance (**a**) the next day and (**b**) one week later. Retention performance did not significantly differ for cued and uncued words in both recall sessions. Memory performance before sleep was set to 100%. Values are mean ± SEM.

During the last learning trials prior to sleep, participants correctly remembered 42.89 ± 2.76 German translations of the 92 Dutch words (i.e., 46.62 ± 3.00%). On the morning after sleep, the amount of correctly recalled words did not significantly differ between cued and uncued words (cued: 97.12 ± 2.28%; uncued: 102.81 ± 2.04% with memory performance before sleep set to 100%, *t*(17) = −1.04, *p* = 0.12; see Fig. 2a). As memory for uncued words was greater than for cued words it is unlikely that the lack of a benefit of TMR on memory consolidation during sleep in adolescents is due to low statistical power. We further analyzed “Gain” words (i.e., Dutch words not remembered before sleep but correctly recalled after sleep) and “Loss” words (i.e., words correctly retrieved before sleep but not remembered after sleep) separately. Again, the number of gains and losses did not significantly differ for cued and uncued words (gains: *p* > 0.50; losses: *p* > 0.20). By testing the recall one week later the results did not change meaning that the correctly recalled words did not differ between cued and uncued conditions (cued: 92.08 ± 2.47%; uncued: 96.58 ± 2.94% with memory performance before sleep set to 100%; *t*(17) = −1.66, *p* = 0.31; see Fig. 2b).

**Figure 3:**
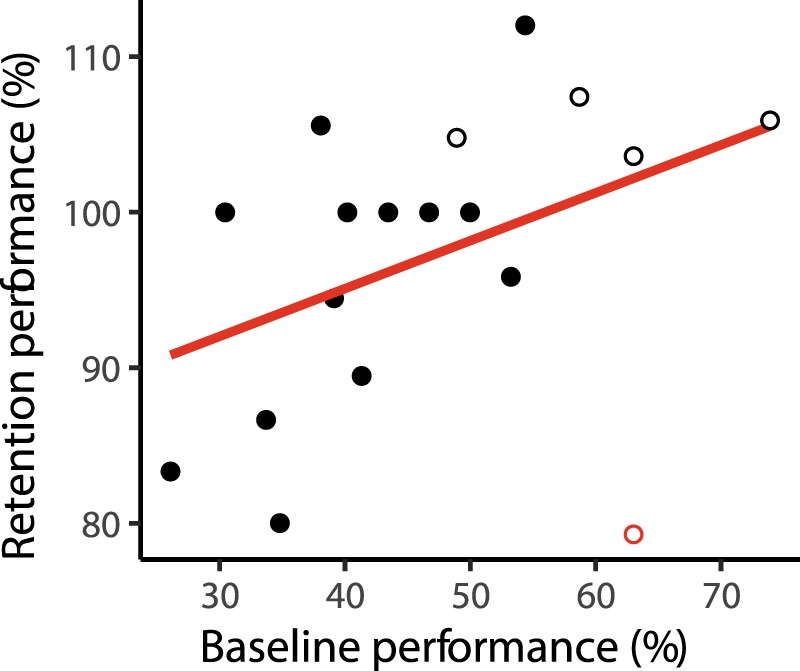
Correlation between baseline and retention performance of cued words. Hollow points represent subjects with one additional learning trial before sleep. Marginal correlation between baseline performance and retention performance (red line, *τ* = 0.35, *p* = 0.046). After exclusion of one outlier (red circle), correlation coefficient further increased (*τ* = 0.50, *p* = 0.006).

Previous studies have reported a relationship between the pre-sleep level of performance and sleep-dependent memory consolidation^7,30–32^. Thus, we also performed correlation analyses between baseline performance (i.e., the relative amount of learnt vocabulary) and retention performance. Baseline performance was significantly correlated with retention performance of cued Dutch words (*τ = *0.35, *p* = 0.046) but not with uncued Dutch words (*τ = *−0.04, *p* = 0.82). The correlation between baseline performance and retention of cued words increased (*τ* = 0.50, *p* = 0.006) after excluding one participant from the analysis who was obviously very distant from other participants (see the red marked data point in Fig. 3). Excluding all participants who received an additional learning round with feedback before sleep yielded similar results. However, by removing these participants we also reduce the sample size. Thus, although the correlation is not significant in this case, it shows a statistical trend (*τ* = 0.38, *p* = 0.078, see hollow marked data points in Fig. 3). Moreover, adding baseline memory performance as a covariate to our main analysis did not change the result pattern (*p* = 0.09), which could be a consequence of a lack of power.

### Neuronal correlates of memory cueing during sleep

**Figure 4:**
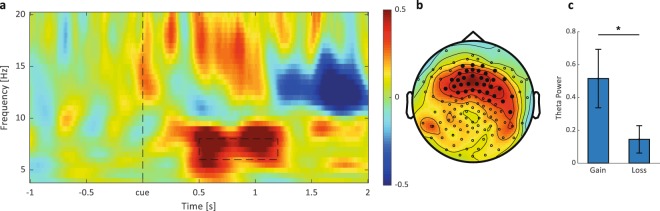
Oscillatory results (n = 14). (**a**) Time-frequency contrasts between Gain and Loss words in the theta band (6–8 Hz) shown for the representative Fz electrode. The dashed box indicates the time (0.5–1.2 s) – frequency (6–8 Hz) area used to illustrate the topographical distribution in (**b**). Significant electrodes are depicted as filled black dots. Subplot (**c**) shows the mean power for Gain and Loss words averaged over time duration, significant channels and theta band (gain: 0.52 ± 0.18; loss: 0.15 ± 0.08; *t*(13) = 2.87, *p* = 0.013). Values are mean ± SEM. *p < 0.05.

In our previous studies in adults we found an increase in theta-power (5–8 Hz) as well as spindle- power (12–16 Hz) after presenting “Gain” words (i.e., cued Dutch words not remembered before sleep but correctly recalled after sleep) with “Loss” words (i.e., cued words correctly retrieved before sleep but not remembered after sleep). These effects were most pronounced over the fronto-central lobe^7^. In a next step, we therefore analyzed theta and spindle power in response to cue exposure. EEG activity during post-learning sleep was increased in response to gains as compared to losses specifically in the theta frequency range in a significant cluster over the frontal cortex 500 to 1200 ms after cue onset (6 to 8 Hz; gain: 0.52 ± 0.18; loss: 0.15 ± 0.08; *t*(13) = 2.87, *p* = 0.013; corrected for multiple comparisons; see Fig. 4 for a description of significant electrodes and their position over the scalp). Importantly, in contrast to our previous studies, we did not find any significant cluster of increased spindle activity (12 to 16 Hz, 500–1000 ms after cue onset, Fig. 4). Moreover, we neither found any significant cluster in the theta nor spindle range during post-learning sleep in response to HitHit words as compared to MissMiss words.

We additionally calculated mean power values within the significant time window (0.5–1.2 s) of delta (1–4 Hz), theta (6–8 Hz) and spindle (12–16 Hz) activity on Fz, Cz and Pz electrodes and the delta and spindle power over the whole night. None of the power values correlated significantly with the number of overnight gains (all *p* > 0.10).

## Discussion

In the present study, we analyzed the impact of targeted memory reactivation on the consolidation of newly acquired Dutch vocabulary in 11 to 13-year-old participants. In contrast to our previous studies in adults, we could not find any beneficial effects of TMR on recall performance the morning after sleep in this particular age group. However, we found an increase in theta activity over frontal regions which is well in line with the previous studies in adults that had identified this oscillatory pattern as a marker of successful memory reactivation. Nonetheless, no increase in spindle activity was observed, which was previously reported during successful reactivation during sleep in young adults.

According to the system consolidation hypothesis, new memories are spontaneously and repeatedly reactivated in the hippocampus during post-learning periods of NREM sleep^2,33^. These reactivations are thought to stabilize the new memory traces for the long-term and integrate them into our neocortical network of preexisting memories. Slow oscillating activity is crucial to this process as it temporally coordinates neuronal activity in different brain regions and thereby possibly facilitates the transfer of neuronal information^2,34,35^. Although slow oscillating sleep EEG activity is at its maximum at the time of late childhood and early adolescence^14–16^, we did not find any beneficial effect of TMR on memory consolidation in this age group. In comparison with previous studies utilizing TMR in adolescents^36^ (N3 = 32.47%), subjects showed a lower amount of slow wave sleep in the current study. We do not believe that the amount of N3 sleep can explain the lack of TMR benefits on memory, because adults have similar or even lower amounts of N3 sleep^37^ and show robust memory increases by TMR^8^. One could speculate that endogenous processes of reactivation are per se highly efficient at this developmental time, thus preventing any further increase by external stimulation. This appears unlikely as retention rates for cued Dutch words were even lower in our group as compared to the group of adults tested in a previous study (children/adolescents: 97%; adults: 105%)^8^. Moreover, we found beneficial effects of TMR using picture-word associations in another previous study in 9–16 year old participants^36^ which also speaks against a general impossibility to improve processes of memory consolidation using TMR.

Alternatively, task-related factors may exist that determine whether memory consolidation can benefit from TMR or not. Because the main aim of this study was to test the effect of TMR in an adolescent sample, we decided to stick to an almost similar paradigm as in our previous TMR study with adults^8^. Our task differs from previous studies testing novel word learning in children^19,21,22^ as we used semantically related learning material. Therefore, memory performance measures cannot directly be compared as different memory processes might underlie these tasks and different mechanisms might support consolidation of novel and related associations.

Here, we found that the baseline performance before sleep was positively correlated with retention performance after sleep for cued words. These results suggest that the beneficial effect of TMR depends on the baseline performance level, which parallels with our previously found correlation between pre-sleep learning performance and the effect of TMR in adults^8^. The idea that overnight consolidation of new memories is related to pre-sleep learning level is also in line with another previous study in children and adults reporting that the pre-sleep level of performance predicts the extent to which sleep as compared to wakefulness improves later memory recall^31^. We hypothesize that greater difficulties to learn new Dutch vocabulary might originate from less prior knowledge in our sample of adolescents. Indeed, emerging evidence indicates that prior knowledge does not only facilitate learning but also critically impacts processes of memory consolidation^38–41^. Accordingly, we recently reported that prior knowledge does not only increase learning performance but that the existence of prior knowledge is an essential prerequisite for beneficial effects of TMR on memory consolidation, which even occurred when accounting for the pre-sleep learning level^30^. Developmental studies provide further support for an impact of prior knowledge on memory consolidation especially in the context of language learning (see also James and colleagues^18^ for a general discussion of the notion that “the rich get richer” in language learning). For example, Henderson and colleagues exposed children and adults to novel words and found that the extent of integration of these words into existing lexical knowledge over time was strongly predicted by already existing vocabulary knowledge^21^. Dyslexic children with deficits in vocabulary knowledge also showed differences in sleep-dependent memory consolidation. While these children show a similar overnight improvement when compared to healthy controls, slow wave and spindle power was significantly associated with overnight improvement only in healthy controls but not in dyslexic children. These findings led authors to speculate that weaker memory traces formed during encoding results in a more passive role of sleep in long-term memory consolidation^32^. Accordingly, a low performance level at learning in our sample might coincide with less stable memory traces, which in turn prevents access to sleep-dependent processes of memory consolidation (see also the discussion on the role of memory strength in sleep-dependent memory consolidation in^42^). This explanation is well in line with our previous findings in the elderly, where participants had profound difficulties in the acquisition of new vocabulary in the learning session and TMR failed to improve memory consolidation during post-learning sleep^43^. Future research should aim to investigate the interplay between pre-sleep level of performance, access to prior knowledge and the effect of TMR on long-term consolidation of new vocabulary by experimentally manipulating these processes separately. An elegant task to experimentally manipulate prior knowledge in the context of language learning which was found to be suitable in children and adults, has been developed by James and colleagues^19^. In this task, prior knowledge was manipulated by exposing novel words that varied in the number of word form “neighbors”. For example, “peflin” was selected as a novel word having no neighbors while “ballow” was selected having many neighbors such as “bellow”, “wallow”, “ballot”, etc.). This task would also allow modifying pre-sleep level of performance by extending or shortening the amount of learning. Interestingly, while distinct words with less prior knowledge are indeed a disadvantage for learning in children and adults in this task, they were consolidated better in children over a week, showing that the role of prior knowledge for processes of memory consolidation is complex.

With regards to brain oscillations, theta activity was increased in response to gain words as compared to loss words, which is well in line with previous studies in adults^7,24,30,44^. However, in contrast to previous studies we were not able to find an increase in spindle activity. Now the question arises whether the lack of increased spindle activity is related to the absent effect of TMR on the behavioral level. We recently proposed that theta activity reflects the reinstatement of the original memory trace while spindle activity is related to the integration of the new memory trace into the neocortical long-term network of preexisting knowledge and its stabilization^13^. According to this theoretical model, both neuronal oscillations are necessary for beneficial effects of TMR on later recall performance. In our study, TMR might have stimulated the reactivation of the newly formed memory traces in the hippocampus as reflected in an increase in theta activity. However, due to low activation of neocortical networks of prior knowledge during learning, neocortical memory traces were too weak to be reactivated by TMR. Consequently, the integration of memory traces into the neocortex and their stabilization was not further improved by TMR, which is reflected by the lack of increased spindle activity. Also other studies have found specific neural correlates of TMR without behavioral effects^34,44,45^. As the tasks in all of these studies, including the current paradigm, previously produced TMR benefits on memory, we believe that other factors (e.g. baseline performance) aside from the task itself explain the lack of memory benefits. Future studies should assess the behavioral effects and the neuronal correlates of reactivating new memories with and without prior knowledge at different pre-sleep learning levels. The combined patterns of behavioral and neurophysiological results will help to uncover relevant factors that determine whether memory gains access to processes of sleep-dependent memory consolidation and it will provide information on underlying neurophysiological mechanisms.

Our findings point towards age-dependent differences in sleep-dependent consolidation of newly acquired memories. We could show that experimentally manipulating processes of memory reactivation during sleep by TMR is feasible in adolescents and can help to increase our understanding about general processes of long-term memory consolidation. Future studies should proceed by applying this technique in order to gain a deeper insight into important factors that facilitate long-term memory consolidation. Importantly, there is a need for larger sample sizes (as compared to the current experiment) in order to take into account the great variability regarding sleep and learning processes in this age-group. Such experiments could have direct implications for educational settings. More specifically, in education, there is a general tendency to teach more in less time. Our findings suggest that more time and more effort lead to improved learning and that more stable memory traces would increase the chance of a new memory content to get stored for the long-term – leading to an ultimately better performance in the long term.

## Data Availability

The datasets generated during the current study are available from the corresponding author on reasonable request.
